# System versus traditional approach in road traffic injury prevention: a call for action

**DOI:** 10.5249/jivr.v3i2.128

**Published:** 2011-07

**Authors:** Davoud Khorasani-Zavareh

**Affiliations:** ^a^Department of Public Health, Urmia University of Medical Sciences, Urmia, Islamic Republic of Iran

Road traffic injuries (RTIs) are a major public health problem worldwide, especially in low- and middle income countries (LMICs) and require concerted efforts for effective and sustainable prevention. A variety of measures need to be considered when planning activities^1-2^Moreover stakeholders’ perceptions,^3-4^approach and the kind of preventive activities are crucial.^2,6^On the whole, there are two different approaches in RTI prevention: the individual approach and the system approach.^2^

In the individual approach, usually there is a tendency for practitioners and researchers to identify only one or a few elements, which usually can be found in many LMICs.^2^Traditionally, in such countries many studies have focused on human errors, poor vehicles design and the road environment rather than focusing on the reason for injury outcome.^6^In many LMICs, the majority of preventive activities target road-user behaviors, which are usually tackled by means of education and enforcement.^2,4^Hence the primary responsibility is assigned to the road user. However, while safe road-user behavior is one important component, changing such behavior should not simply be focused on education and enforcement.^2,3^Studies on public education efficiency have revealed that a decrease in collisions due to such campaigns can be occurred only if they clearly focus on specific forms of behavior, like seat belt use or helmet wearing.^4^

In contrast, a system approach tends to be mainly directed toward the crashworthiness of the road transport system.^2,5^For example, Sweden has been rather successful in this area and one major national policy is a long-term vision for road safety, “Vision Zero”. It was a revolutionary way of thinking about traffic user safety that helped Sweden to significantly reduce the number of deaths and serious injuries due to road traffic crashes. This is a road safety policy that puts the protection of the most vulnerable road-users at its centre.^6^The system designer has the primary responsibility and as a result changes within the environment are given more emphasis than human factors.^5^The road transport system should be able to consider human failings and absorb errors in the road transport system, in order to avoid serious RTIs and deaths. It is interesting to note that the “Vision Zero” is not just applicable to high-income countries and it could be transferred to low- and middle-income countries. Accordingly, in such countries, if the inherent safety of the system (road and vehicle safety design) cannot be changed, then the only way to reduce RTIs is to lower speeds, as a means of speed management. The basic principles of Vision Zero can be used in any type of road transport system, at any stage of development.^6^
